# 2298 Central autonomic network dysfunction implicated in alcohol-related intimate partner violence

**DOI:** 10.1017/cts.2018.52

**Published:** 2018-11-21

**Authors:** Brandi C. Fink

**Affiliations:** Clinical and Translational Science Center, University of New Mexico

## Abstract

OBJECTIVES/SPECIFIC AIMS: Most incidents of partner violence occur when one or both partners have been drinking, however, the mechanism through which this association exists is unclear. The neural circuits that support self-regulation of emotion and social behavior, as well as autonomic influences on the heart, are co-localized in the brain and represent an integrated bidirectional regulatory system. These physiological regulatory processes are mediated by a neural substrate known as the central autonomic network which includes the peripheral autonomic nervous system. The central autonomic network modulates biobehavioral resources in emotion by flexibly responding to physiological arousal in response to changing situational demands, and serves a fundamental role in emotion regulation and goal-directed motor behavior, and this circuit can be indexed with heart rate variability (HRV). METHODS/STUDY POPULATION: In total, 17 distressed violent (DV) partners (11 females, 6 males) were matched to a sample of distressed nonviolent (DNV) partners (7 female, 6 males) were matched on age, sex, and relationship satisfaction and participated in a placebo-controlled alcohol administration study with an emotion-regulation task during which electroencephalography, HRV, and galvanic skin response (GSR) measures were collected. In the alcohol condition, participants were administered a mixture of 100 proof vodka and cranberry juice calculated to raise their blood alcohol concentration to 0.08%. In the placebo condition, participants consumed a volume of juice equivalent to that consumed in the alcohol condition, but without alcohol. Alcohol and placebo conditions were counter-balanced across participants as were the presentation the blocks of evocative and neutral partner stimuli and emotion-regulation condition (watch vs. do not react). RESULTS/ANTICIPATED RESULTS: Results show that DV partners show greater cortical arousal than DNV partners on measures event-related spectral perturbations, which are mean log event-loced deviations from baseline-mean power at each frequency of the electroencephalography power spectra, when intoxicated and viewing evocative partner stimuli in the “do not react” emotion regulation condition. Results also show a statistically significant 2 (alcohol vs. placebo)×2 (watch vs. do not react)×2 (DV partners vs. DNV partners) interaction of the respiratory sinus arrhythmia measure of HRV when viewing evocative partner behavior (*F*=7.102, *p*=0.019, partial η^2^=0.353). Findings indicate that DV partners have lower HRV than DNV partners across conditions, but particularly when acutely intoxicated and trying not to react to their partners’ evocative behavior. Similarly, results also show a statistically significantly 2 (alcohol vs. placebo)×2 (watch vs. do not react)×2 (DV partners vs. DNV partners) interaction on GSR (*F*=71.452, *p*=0.000, partial η^2^=0.749). GSR findings indicate that DV partners also have lower GSR when acutely intoxicated and trying not to react to their partners’ evocative behavior. DISCUSSION/SIGNIFICANCE OF IMPACT: These results suggest that increases in intimate partner violence under acute alcohol intoxication may be the result of dysfunction of the central autonomic network, especially when DV partners are trying to suppress a behavioral response to their partners’ evocative behavior in conflict. The neurophysiological patterns evidenced by DV partners is consistent with a state of vigilance to threat, and reduced ability inhibit prepotent, but inappropriate responses. They also suggest that HRV may be an important target for intervention with partner with a history of intimate partner violence. One method may be heart rate variability biofeedback which has been shown to increase parasympathetic nervous system functioning, autonomic stability, and emotion regulation.

